# Remnant preservation technique versus standard technique for anterior cruciate ligament reconstruction: a meta-analysis of randomized controlled trials

**DOI:** 10.1186/s13018-018-0937-4

**Published:** 2018-09-12

**Authors:** Hong-De Wang, Fu-Shun Wang, Shi-Jun Gao, Ying-Ze Zhang

**Affiliations:** 1 Department of Orthopaedic Surgery, The Third Hospital of Hebei Medical University, No. 139 Ziqiang Road, Qiaoxi District, Shijiazhuang, 050051 People’s Republic of China; 2 Department of Orthopaedic Surgery, XinHuaFuShun Clinic of Traditional Chinese and Western Medicine, No. 398 Youyi North Street, Xinhua District, Shijiazhuang, 050051 People’s Republic of China; 3 Key Laboratory of Biomechanics of Hebei Province, Shijiazhuang, 050051 Hebei People’s Republic of China; 4 Chinese Academy of Engineering, Beijing, 100088 People’s Republic of China

**Keywords:** Anterior cruciate ligament, Reconstruction, Remnant preservation, Meta-analysis

## Abstract

**Background:**

This meta-analysis was performed to compare the clinical outcomes of primary anterior cruciate ligament (ACL) reconstruction using the ACL remnant preservation technique versus the standard technique.

**Methods:**

PubMed, Embase, and the Cochrane Library were searched through December 24, 2017, to identify randomized controlled studies that compared the use of the ACL remnant preservation technique versus the standard technique for primary ACL reconstruction. Statistical heterogeneity among the trials was evaluated with chi-square and *I*-square tests. A sensitivity analysis was conducted to explore sources of heterogeneity. Subgroup analysis was performed to identify potential differences according to type of ACL remnant tissue (remnant bundle or remnant fibers).

**Results:**

Seven studies with a combined 412 patients (208 in the remnant preservation technique group and 204 in the standard technique group) were included in the meta-analysis. There was a significant difference between the groups in Lysholm score (mean difference (MD), 2.20; 95% confidence interval (CI), 0.95–3.45; *P* = 0.0006) and side-to-side difference (MD, − 0.71; 95% CI, − 0.87 to − 0.55; *P* < 0.01). There was no significant difference between the groups in subjective International Knee Documentation Committee (IKDC) score, complications, pivot shift test, Lachman test, or overall IKDC score. Subgroup analysis demonstrated that for primary ACL reconstruction with preservation of remnant fibers, the remnant preservation technique was superior to the standard technique based on Lysholm scores (*P* < 0.01) and side-to-side difference (*P* < 0.01).

**Conclusions:**

Based on the current literature, using the remnant preservation technique showed a better clinical outcome than using the standard technique for patients undergoing primary ACL reconstruction with respect to Lysholm score and side-to-side difference. However, it remains unclear that there is a definite advantage to use the remnant preservation technique compared with the standard technique.

**Electronic supplementary material:**

The online version of this article (10.1186/s13018-018-0937-4) contains supplementary material, which is available to authorized users.

## Background

Anterior cruciate ligament (ACL) injury is one of the most common knee injuries with an annual incidence of 68.6 per 100,000 person-years [1, 2]. An injured ACL cannot heal naturally and will lead to an increased risk of meniscal injury and osteoarthritis [3–5]. Thus, ACL reconstruction is a conventional surgical technique to restore function to the knee with a ruptured ACL, and excellent clinical outcomes have been reported.

Residual ACL remnants are commonly observed during arthroscopic examination. To identify the ACL attachment, the ACL remnant is debrided clearly during ACL reconstruction using standard techniques. In recent years, the importance of the ACL remnant has been recognized in terms of biomechanical, vascular, and proprioceptive function. Some studies reported that mechanoreceptors that control knee proprioception are located in the inner membrane of the synovium near the tibial attachment of the ACL [6, 7]. In addition, the ACL remnant tissue has good subsynovial and intrafascicular vascularity [6]. This may accelerate cell repopulation and revascularization in the graft. However, the clinical outcomes remain controversial as to the use of the remnant preservation technique versus the standard technique. Some studies reported good clinical outcomes following remnant preservation [8–10]. Kondo et al. [10] reported that the benefit of the remnant preservation technique can significantly improve postoperative knee stability. Lee et al. [11] reported that patients with a remnant greater than 20% of the length of the ACL had better proprioceptive function than those with less than 20% length. Conversely, some authors have reported that there is no significant difference between the two techniques, and even that remnant preservation may increase the risk of certain complications and subsequently affect the functional performance of the knee [12, 13].

Based on the current evidence, the purpose of this meta-analysis of randomized controlled trials (RCTs) on the clinical outcomes following either the remnant preservation or the standard technique of ACL reconstruction was to lead to the appropriate selection of technique to provide the greatest benefit to patients. The clinical outcomes that were assessed included knee functionality, stability, subjective evaluation, and complication rate.

## Methods

This meta-analysis was performed according to the guidelines outlined in the Preferred Reporting Items for Systematic Reviews and Meta-Analyses (PRISMA) statement [14].

### Study eligibility

Two reviewers independently decided which studies to include based on the selection criteria. The inclusion criteria were as follows: (1) the article described a RCT (level of evidence, I or II), (2) only the isolated ACL injury, (3) primary ACL reconstruction was performed, (4) reconstruction was performed with the remnant preservation technique or the standard technique, and (5) the study included clinically relevant subjective and objective outcomes, such as subjective patient evaluation, complications, stability, and function.

The exclusion criteria were as follows: (1) the article described a case-control study, retrospective cohort study, case series, review article, letter to the editor, or technique note, (2) injury to multiple knee ligaments, meniscal injury, and/or cartilage injury requiring surgery, (3) the study included the same patients from the same center undergoing the same technique with different follow-up intervals.

### Literature search

We searched PubMed, Embase, and the Cochrane Library to identify RCTs published from the initial date to 24 December 2017 that compared the remnant preservation technique with the standard technique for primary ACL reconstruction. The title and abstract fields were searched for the following terms in each database: anterior cruciate ligament, remnant, preservation. A manual search was also performed for articles potentially missed by the electronic search. The search history of each database is supplied in Additional file 1, Additional file 2, and Additional file 3.

### Study selection and data extraction

Two reviewers independently decided which studies to include based on the selection criteria. Studies were selected in two levels of screening: screening of the titles and abstracts and screening of the full texts. Disagreement between the reviewers was resolved by consensus or by discussion with the senior author if a consensus could not be reached.

The extracted data were assessed by two independent reviewers who reviewed basic information including first author, publication year, study type, sample size, mean age, sex ratio, graft type, fixation method, mean follow-up, and quality assessment score in standardized forms. The primary outcomes were subjective patient evaluation, including subjective International Knee Documentation Committee (IKDC) score and Lysholm score, and complications. The secondary outcomes were knee stability, including the pivot shift test, Lachman test, and side-to-side difference, and knee function, including overall IKDC score. The side-to-side difference was measured with a KT-1000/2000 arthrometer. The mean and standard deviation were not reported in some studies and were calculated by using statistical formulas if the related data was provided [15]. Disagreements were resolved by discussion among the authors.

### Risk of bias assessment

Two authors independently graded the methodological quality of each eligible study using the Cochrane Collaboration tool to assess the risk of bias for RCTs [16]. The authors assessed random sequence generation, allocation concealment, blinding of participants and personnel, blinding of outcome assessors, incomplete outcome data, selective outcome reporting, and other bias (baseline balance and funding). All fields were judged as having a low risk of bias, high risk of bias, or unclear risk of bias.

### Data analysis

Data analysis was performed with RevMan (Version 5.3; Copenhagen: The Nordic Cochrane Centre, The Cochrane Collaboration, 2014). A random-effects model was adopted to pool the results. The risk ratio (RR) was used as a summary statistic for dichotomous variables, and the mean difference (MD) was used to analyze continuous variables. Both were reported with 95% confidence intervals (CIs), and a *P* value of 0.05 was used as the level of statistical significance. Statistical heterogeneity between trials was evaluated with the chi-square and *I*-square tests (*I*^2^: 0–30% was considered homogeneity, 30–60% was considered moderated heterogeneity, and > 60% was considered substantial heterogeneity), with significance set at *P* < 0.10. A sensitivity analysis was conducted to explore sources of heterogeneity. We also performed a subgroup analysis to identify potential differences according to the type of ACL remnant tissue (remnant bundle or remnant fibers).

## Results

### Characteristics of included studies

**Fig. 1:**
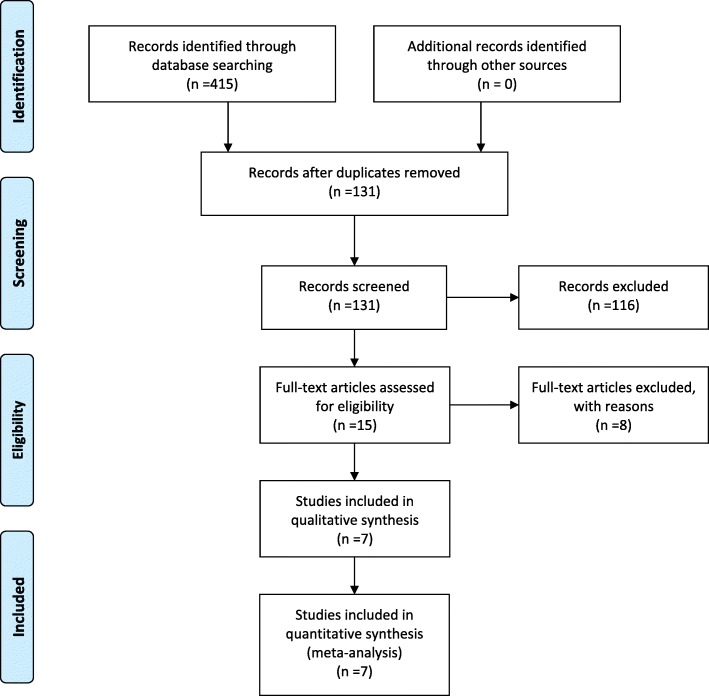
Selection process for the meta-analysis of studies comparing the ACL remnant preservation technique with the standard technique for ACL reconstruction

A summary of the study selection process is presented in Fig. 1. Our search identified 415 records. A total of 284 citations were discarded because they were duplicates or did not fit the eligibility criteria. After full-text verification of the remaining 15 articles, seven studies with a combined 412 patients (208 in the remnant preservation technique group and 204 in the standard technique group) were included in the meta-analysis [17–23].

All included studies randomized patients to primary ACL reconstruction using hamstring tendon autografts or allografts. All seven studies used the same fixation method for both the remnant preservation technique group and the standard technique group. However, the fixation methods differed between studies. Femoral fixation was obtained with an EndoButton in three studies, RigidFix in two studies, Cross-pin in one study, and Interference screw in one study. Tibial fixation was obtained with a bioabsorbable interference screw in six studies and with an Intrafix in one study. Among the 412 patients, 404 patients used soft-tissue grafts including hamstring tendons and tibialis anterior tendons for ACL reconstruction. Only eight patients used bone-patellar tendon-bone grafts in the study of Pujol et al. Both of the two groups of patients received the same rehabilitation protocols in each included RCT. The characteristics of the included studies are shown in Table 1.

**Table 1:** Characteristics of included studies

Study	Year	Study type	Sample (RP/ST)	Mean age (RP/ST)	Gender M,F (RP/ST)	Surgical technique	Graft type	Fixation method (F/T)	Follow-up interval (mo)(RP/ST)	Quality assessment^a^
Andonovski et al.	2017	RCT	33/33	28/28	NR/NR	A, SB	HT	Endobutton/Interfernce screw	7/7	Unclear risk
Lu et al.	2015	RCT	36/36	29.3/31.4	36,0/36,0	A, DB	HT	Endobutton/Interfernce screw	34.7/39.6	Unclear risk
Hong et al.	2012	RCT	39/41	31/31	33,12/34,11	A, SB	TA/HT allograft	RigidFix/IntraFix	25.8/25.5	Unclear risk
Pujol et al.	2012	RCT	29/25	31.24/28.56	16,13/17,8	A, SB	HT/BPTB	Interference screw, cortical button/Interference screw, double fixation	12/12	Unclear risk
Demirağ et al.	2012	RCT	20/20	31/28	18,2/18,2	A, SB	HT	Cross-pin/Screw	24.3/24.3	Unclear risk
Zhang et al.	2014	RCT	27/24	23.5/25.3	19,4/21,5	A, SB	HT	RigidFix/Interference screw	24.4 ± 25.2	Unclear risk
Gohil et al.	2007	RCT	24/25	30.5/35.5	14,10/13,12	A, SB	HT	Endobutton/Interfernce screw	12/12	Unclear risk

RCT, randomized controlled trial; M, male; F, female; mo, month; NR, not reported; RP, remnant preservation; ST, standard; A, anatomic reconstruction; SB, single-bundle reconstruction; DB, double-bundle reconstruction; HT, hamstring tendon; TA, tibialis anterior; BPTB, bone-patellar tendon-bone

^a^Cochrane Collaboration Risk of Bias for RCTs (graded as low risk of bias, high risk of bias, or unclear risk of bias)

### Meta-analysis of clinical outcomes

#### Primary outcomes

**Fig. 2:**
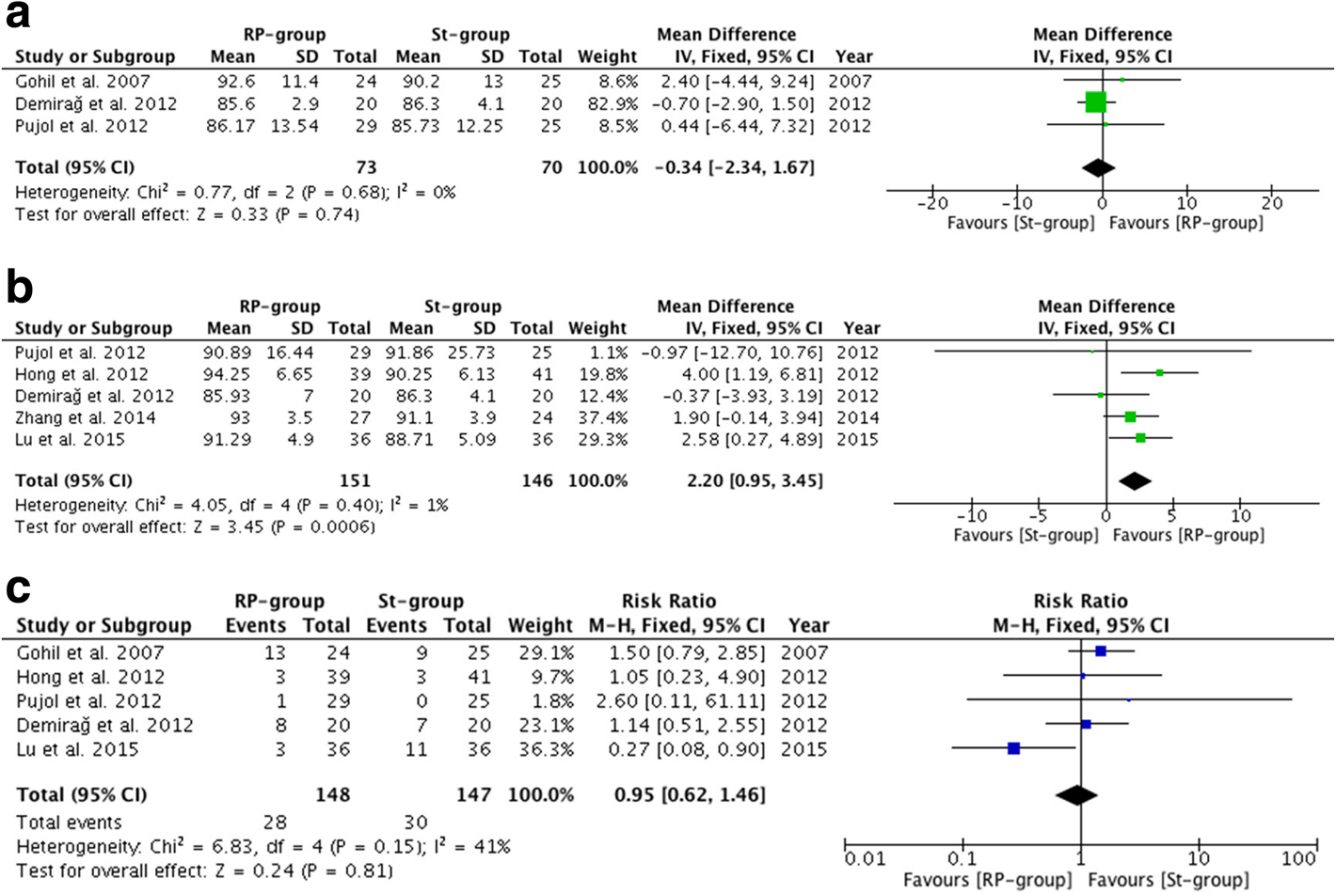
Primary outcomes after anterior cruciate ligament (ACL) reconstruction. RP-group, Remnant preservation technique group; St-group, Standard technique group. **a** Subjective International Knee Documentation Committee scores after ACL reconstruction. **b** Lysholm scores after ACL reconstruction. **c** Complications after ACL reconstruction

The results of the Lysholm scores (MD, 2.20; 95% CI, 0.95–3.45; *P* = 0.0006) (*P* = 0.40 and *I*^2^ = 1% for heterogeneity) showed a statistically significant difference between the remnant preservation technique and the standard technique in favor of the remnant preservation technique. There was no significant difference between the groups in subjective IKDC scores (MD, − 0.34; 95% CI, − 2.34–1.67; *P* = 0.74) (*P* = 0.68 and *I*^2^ = 0% for heterogeneity) or complications (RR, 0.95; 95% CI, 0.62–1.46; *P* = 0.81) (*P* = 0.15 and *I*^2^ = 41% for heterogeneity). The results of the primary outcomes are illustrated in Table 2 and Fig. 2. In addition, there was no significant difference between the groups in any individual complication (including revision rate, cyclops lesion or arthrofibrosis) except for impingement (RR, 0.50; 95% CI, 0.30–0.84; *P* = 0.009). The individual complication results are illustrated in Table 3.

**Table 2:** Clinical outcomes

	Number of included studies	Number of included patients	MD/RR	95% CI	Heterogeneity (*P*/*I*^2^)	*P* value
Primary outcomes						
Subjective IKDC	3	143	MD −0.34	[−2.34, 1.67]	0.68/0%	0.74
Lysholm score	5	297	MD 2.20	[0.95, 3.45]	0.40/1%	0.0006
Complications	5	295	RR 0.95	[0.62, 1.46]	0.15/41%	0.81
Secondary outcomes						
Pivot shift test	4	246	RR 1.06	[0.97, 1.17]	0.80/0%	0.20
Lachman test	2	120	RR 1.04	[0.87, 1.23]	0.81/0%	0.69
Side-to-side difference	4	269	MD −0.71	[−0.87, −0.55]	< 0.01/91%	< 0.01
Overall IKDC	3	206	RR 1.05	[0.96, 1.14]	0.34/8%	0.27

IKDC, International Knee Documentation Committee

**Table 3:** Complications

	Number of included studies	Number of included patients	RR	95% CI	Heterogeneity (*P*/*I*^2^)	*P* value
Revision	1	72	0.20	[0.01, 4.03]	–	0.29
Cyclops lesion	4	223	1.51	[0.84, 2.70]	0.92/0%	0.17
Arthrofibrosis	1	40	1.00	[0.43, 2.33]	–	1.00
Impingement	1	72	0.50	[0.30, 0.84]	–	0.009

#### Secondary outcomes

**Fig. 3:**
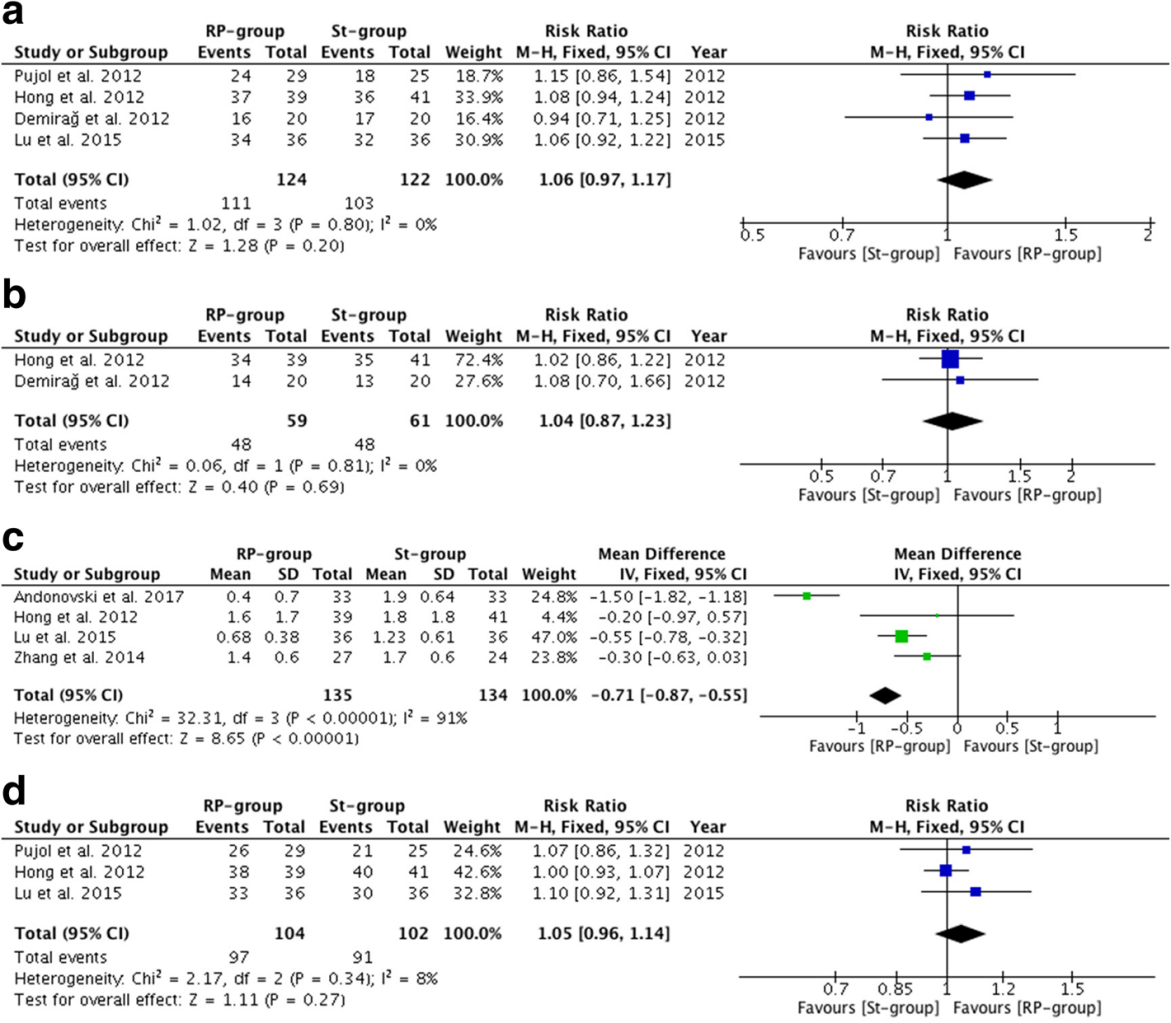
Secondary outcomes after anterior cruciate ligament (ACL) reconstruction. RP-group, Remnant preservation technique group; St-group, Standard technique group. **a** Pivot-shift test (Grade 0) after ACL reconstruction. **b** Lachman test (Grade 0) after ACL reconstruction. **c** Side-to-side difference after ACL reconstruction. **d** Overall International Knee Documentation Committee score (Normal, Nearly normal) after ACL reconstruction

Except for side-to-side difference (MD, − 0.71; 95% CI, − 0.87−− 0.55; *P* < 0.01) (*P* < 0.01 and *I*^2^ = 91% for heterogeneity), which was in favor of the remnant preservation technique, there was no significant difference between the remnant preservation technique and the standard technique with respect to secondary stability outcomes, including the pivot shift test and the Lachman test. Moreover, there were no significant differences in secondary functional outcomes on overall IKDC scores. The secondary outcome results are illustrated in Table 2 and Fig. 3.

### Sensitivity analysis and subgroup analysis

Sensitivity analysis was conducted to explore the possibility of heterogeneity in stability outcomes. The results showed that there was no particularly influential study among the included studies, except for the effects of the studies of Andonovski on side-to-side difference [23]. The mean follow-up time in this trial was 7 months. Exclusion of this trial did not alter the results of the side-to-side difference. (MD, − 0.45; 95% CI, − 0.64 to − 0.26; *P* < 0.01) (*P* = 0.39 and *I*^2^ = 0% for heterogeneity).

Subgroup analysis was performed according to type of remnant tissue (remnant bundle or remnant fibers), as displayed in Table 4. Significant differences were found in both subgroups between the remnant preservation technique and the standard technique in side-to-side difference, and in the subgroup of remnant fibers between the remnant preservation technique and the standard technique in Lysholm scores.

**Table 4:** Results of subgroup analysis

	Remnant bundle				Remnant fibers			
	MD/RR	95% CI	Heterogeneity (*P*/*I*^2^)	*P* value	MD/RR	95% CI	Heterogeneity (*P*/*I*^2^)	*P* value
Primary outcomes								
Subjective IKDC	MD −0.59	[−2.69, 1.50]	0.76/0%	0.58	MD 2.40	[−4.44, 9.24]	–	0.49
Lysholm score	MD −0.42	[−3.82, 2.98]	0.92/0%	0.81	MD 2.61	[1.27, 3.96]	0.49/0%	< 0.01
Complication	RR 1.25	[0.57, 2.73]	0.61/0%	0.58	RR 0.85	[0.51, 1.42]	0.04/70%	0.54
Secondary outcomes								
Pivot shift test	RR 1.05	[0.85, 1.29]	0.34/0%	0.63	RR 1.07	[0.97, 1.18]	0.87/0%	0.16
Lachman test	RR 1.08	[0.70, 1.66]	–	0.74	RR 1.02	[0.86, 1.22]	–	0.81
Side-to-side difference	MD −1.50	[−1.82, −1.18]	–	< 0.01	MD −0.45	[−0.64, −0.26]	0.39/0%	< 0.01
Overall IKDC	RR 1.07	[0.86, 1.32]	–	0.55	RR 1.04	[0.96, 1.14]	0.28/45%	0.35

IKDC, International Knee Documentation Committee

### Risk of bias in the included studies

**Fig. 4:**
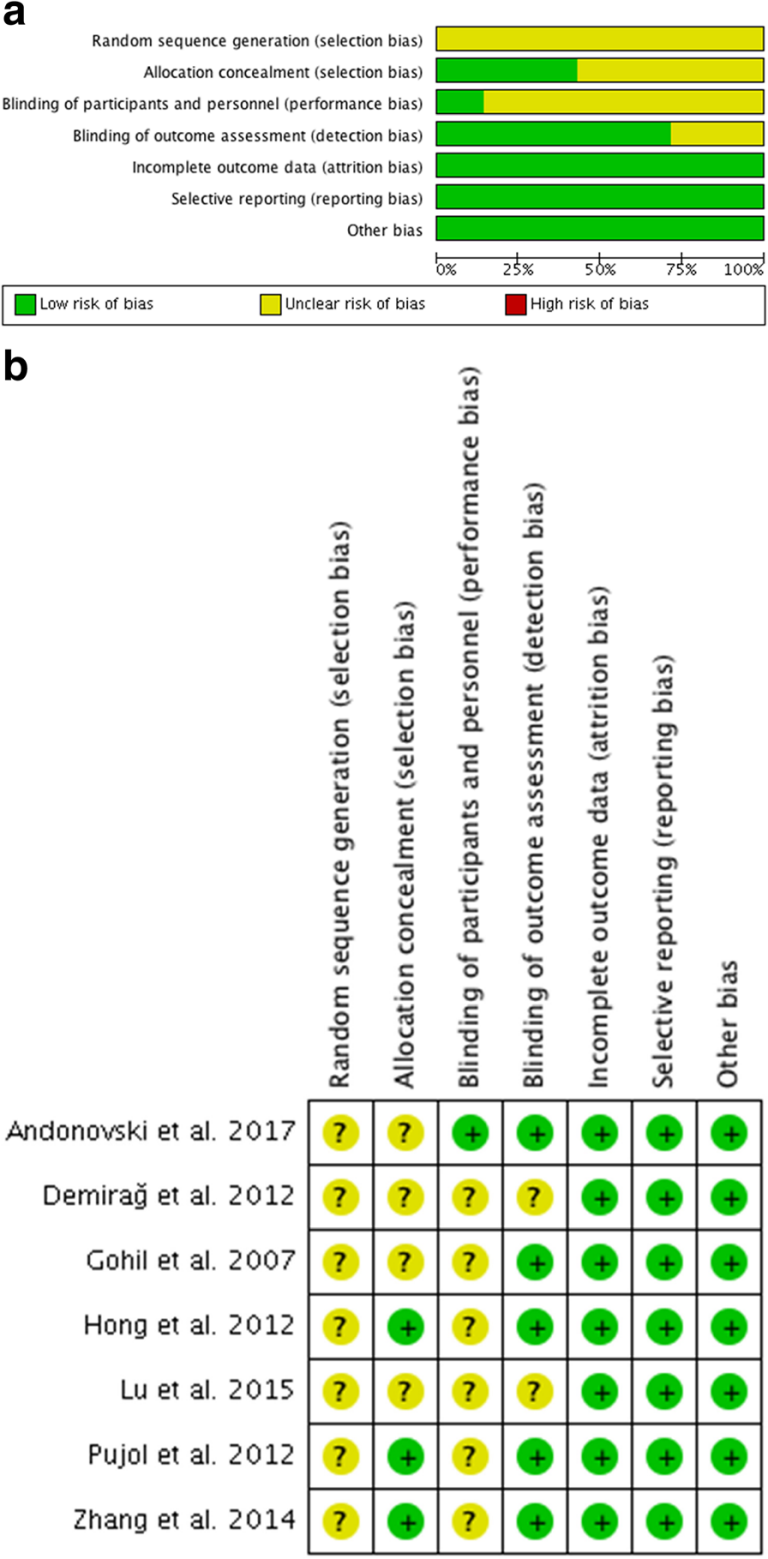
Risk of bias for each randomized controlled trial (RCT). **a** Graph depicting risk of bias. **b** Summary of risk of bias in the included studies

Information about the risk of bias in each study is presented in Fig. 4. All seven studies had an unclear risk of bias. Random sequence generation was not reported in these seven studies. All of these studies lacked blinding of the participants except for the study by Andonovski. Allocation concealment was carried out adequately in three studies.

## Discussion

This meta-analysis that included seven RCTs suggested that the remnant preservation technique during ACL reconstruction showed a better clinical outcome compared with the standard technique with respect to Lysholm score and side-to-side difference.

Remnant preservation has been recognized to have an important role in ACL reconstruction. However, its actual effectiveness remains controversial. For patients with ACL injury, the first concern is subjective patient evaluation and complications. The differences in subjective evaluation and complications between the use of the remnant preservation technique and the standard technique play an important role in patient choice.

Some previous studies reported that there are mechanoreceptors located in the subsynovial layer near the tibial site of ACL fibers [24, 25]. Moreover, many authors showed that the regeneration of mechanoreceptors may be accelerated by revascularization of the graft and that the recovery of knee proprioceptive function could be promoted by the surviving mechanoreceptors of the ACL remnant and the regenerated mechanoreceptors [11, 25–29]. Therefore, some authors have preserved the ACL remnant during ACL reconstruction, assuming that remnant preservation can better restore proprioceptive and functional outcomes of the knee joint [8, 10, 11, 27, 30–32]. Kondo et al. [10] reported that remnant preservation significantly improved postoperative knee stability and arthroscopic evaluation than using the remnant resecting technique during ACL reconstruction. Lee et al. [11] reported in a group of 16 patients that better proprioceptive and functional outcomes occurred in those with a preserved remnant greater than 20% of the length of the ACL than in those where the remnant was less than 20%. Yanagisawa et al. [8] reported that the remnant preservation technique reduces the amount of bone tunnel enlargement. Kitamura et al. [32] demonstrated that the preservation of ACL remnant tissue in anatomic double-bundle ACL reconstruction appears to improve the control of pivot-shift laxity at a minimum of 12 months after surgery. However, one study that was included in our analysis demonstrated that remnant preservation had no significant advantage over the standard technique in terms of proprioception function. The other six RCTs included in our analysis did not assess this outcome, so the proprioception between the two techniques cannot be compared. In addition, the proprioceptive function of the knee joint does not depend solely on the ACL. Other factors such as muscles, ligaments, menisci, the joint capsule, and even skin can influence proprioception and make it difficult to directly compare the studies. Therefore, more accurate measurements and clinical outcome scores should be introduced to assess proprioceptive function.

The remnant ACL tissue has good subsynovial and intrafascicular vascularity [6]. Wu et al. [33] reported in an experimental study that blood flow to the grafts was significantly higher in the remnant-preserved group than in the remnant-resected group. Therefore, many authors believed that as the remnant was preserved, a portion of blood vessels from the tibial attachment were also preserved, which may accelerate cell repopulation and revascularization in the graft, resulting in acceleration of graft remodeling and early restoration of the mechanical properties of the graft [34] [35–37]. Ahn et al. [34] reported that magnetic resonance imaging showed significantly larger ACL grafts in the remnant bundle preservation group than in the standard procedure group, and these preserved remnant bundles showed progressive remodeling in the ACL graft. In addition, improved graft remodeling was confirmed by using arthroscopic second-look evaluation [27, 31]. Ahn et al. [27] performed a second-look evaluation in 62% patients who underwent ACL reconstruction with remnant preservation and reported that 91% had fair synovialization of the ACL graft. Kondo et al. [10] demonstrated on second-look evaluation that the remnant-preserving procedure was significantly better than the remnant-resecting procedure with regard to postoperative laceration or tear of the grafts as well as synovial and fibrous tissue coverage of the grafts. Two RCTs included in our study reported the second-look evaluation [20, 22]. Lu et al. [22] showed that the grafts in the remnant preservation group had a better quality in terms of synovium coverage, apparent tension, and thickness compared with the standard group. However, Hong et al. [20] observed no significant difference between the two groups on second-look evaluation. Allograft was used in Hong’s study, and the follow-up time was 26 months. Previous studies demonstrated that the incorporation of allografts was delayed and that complete remodeling and cellular replacement of the entire graft may require 3 years or longer [38, 39]. This may be a potential cause for increased side-to-side difference especially in the early follow-up. Thus, the graft type (autografts or allografts) and the short follow-up time may have contributed to no significant difference being observed on second-look evaluation between the two groups.

Several studies confirmed that preservation of the remnant during ACL reconstruction can influence the stability of the knee joint, particularly the anterior-posterior stability [27, 30, 40]. Adachi et al. [40] reported that KT-1000 results in remnant-preserved group were 0.7 ± 1.8 mm versus 1.8 ± 2.1 mm in the standard technique group (*P* < 0.05). Kim et al. [11] reported that the postoperative mean side-to-side difference was 1.67 mm on KT-2000 in patients who underwent double-bundle ACL reconstruction with the remnant-preserved technique. According to our meta-analysis, the side-to-side difference results in both groups were comparable to previous cohort studies. There are likely several reasons why postoperative knee stability was significantly improved by sufficient preservation of the ACL remnant tissue. The biology of graft healing is a process of creeping substitution [36, 37]. The ACL remnant has good subsynovial and intrafascicular vascularity. Therefore, first, the preserved remnant may accelerate the revascularization and ligamentation of the grafts, as well as its incorporation and stability. Second, the present study showed that in remnant-preserved reconstruction, the tibial attachment of the ACL remnant tissue, which appeared to be almost normal, was maintained around the tibial tunnel site. In contrast, Tomita et al. [41] reported that the tibial attachment in the remnant-resected ACL reconstruction was narrow and had a different shape in comparison with the normal attachment. This may explain the finding that the impingement rate was significantly higher in the standard technique group compared with the remnant preservation group (*P* = 0.009).

A potential complication of ACL reconstruction using remnant preservation is cyclops syndrome or a cyclops lesion [28]. The incidence of cyclops syndrome, which involves serious loss of knee extension caused by a hard nodule around the reconstructed ACL, has been reported to range from 2 to 11% [42, 43]. The incidence of a cyclops lesion, which is a soft synovial tissue mass without any clinical symptoms around the reconstructed ACL, has been reported to range from 2 to 47% [27, 42, 44]. Four RCTs included in our meta-analysis reported cyclops lesion occurrence (18 of 112 in the remnant preservation technique group, 12 of 111 in the standard technique group) [17, 19–21]. Only one patient required arthroscopic arthrolysis for cyclops syndrome [21]. Some magnetic resonance imaging studies showed that there was no significant difference in the prevalence of cyclops lesion after single-bundle ACL reconstruction between the remnant-preserved and remnant-resected techniques (12.2% and 15.0%, respectively). The results of our meta-analysis showed no significant difference in the occurrence of cyclops lesions between the remnant preservation technique and the standard technique (16.1% and 10.8%, respectively. *P* = 0.17). Thus, we hypothesized that the preservation of ACL remnant tissue does not increase the incidence of cyclops lesions.

The types of remnant preservation used in the seven RCTs were either remnant bundle preservation or remnant fiber preservation. The former type can be defined as a single-bundle rupture (anteromedial bundle or posterolateral bundle) with the other bundle remnant preserved. The latter type can be defined as a double-bundle rupture with the remnant fiber preserved. According to the results of the subgroup analysis, there were significant differences in terms of side-to-side difference between the subgroup and the standard technique group. This finding suggests that if the remnant tissue is a bundle, surgeons should attempt to preserve the remnant bundle, while if the remnant tissue is only fibers, the remnant fibers should be preserved. Interestingly, there was a significant difference in Lysholm scores between the subgroup of remnant fiber preservation and the standard technique group in favor of the subgroup.

### Limitation

This study had several limitations. First, all seven studies were rated as having an unclear risk of bias because the method of blinding patients was not reported or blinding was not used. Blinding is rarely possible in surgical studies, which is an inherent limitation of conducting randomized trials. Second, the standard deviation was unavailable in some studies, so the imputed standard deviation was used for pooling of the data. Third, the duration of final follow-up was substantially different among the included studies, ranging from 7 to 49 months, and this difference may have obscured the reporting of differences between the two groups. Finally, the grafts used for ACL reconstruction were not of the same type and included autograft and allograft, which might influence the incorporation between the remnant and graft.

## Conclusion

Based on the current literature, using the remnant preservation technique showed a better clinical outcome than using the standard technique for patients undergoing primary ACL reconstruction with respect to Lysholm score and side-to-side difference. However, it remains unclear that there is a definite advantage to use the remnant preservation technique compared with the standard technique.

## Additional files

Additional file 1: PubMed. (DOCX 125 kb)

Additional file 2: Embase. (DOCX 63 kb)

Additional file 3: Cochrane. (DOCX 81 kb)

## Abbreviations

ACL: Anterior cruciate ligament
CIs: Confidence intervals
IKDC: International Knee Documentation Committee
MD: Mean difference
PRISMA: Preferred Reporting Items for Systematic Reviews and Meta-Analyses
RCTs: Randomized controlled trials
RR: Risk ratio
