# Supplementary material for: Remnant preservation technique versus standard technique for anterior cruciate ligament reconstruction: a meta-analysis of randomized controlled trials
Source: J Orthop Surg Res. 2018 Sep 12;13:231. doi: 10.1186/s13018-018-0937-4 (PMC6134761; doi:10.1186/s13018-018-0937-4)
Supplement: Supplementary file 1 — PubMed. (DOCX 125 kb) [file 13018_2018_937_MOESM1_ESM.docx]

Recent queries in pubmed

Search,Query,Items found,Time

#13,"Search (((""Anterior Cruciate Ligament""[Mesh]) OR (((((((((((((Anterior cruciate ligament[Title/Abstract]) OR Anterior Cranial Cruciate Ligament[Title/Abstract]) OR Cranial Cruciate Ligament[Title/Abstract]) OR Cranial Cruciate Ligaments[Title/Abstract]) OR Cruciate Ligament, Cranial[Title/Abstract]) OR Cruciate Ligaments, Cranial[Title/Abstract]) OR Ligament, Cranial Cruciate[Title/Abstract]) OR Ligaments, Cranial Cruciate[Title/Abstract]) OR Cruciate Ligament, Anterior[Title/Abstract]) OR Anterior Cruciate Ligaments[Title/Abstract]) OR Cruciate Ligaments, Anterior[Title/Abstract]) OR Ligament, Anterior Cruciate[Title/Abstract]) OR Ligaments, Anterior Cruciate[Title/Abstract]))) AND remnant[Title/Abstract]",194,10:40:08

#12,"Search remnant[Title/Abstract]",16726,10:19:16

#11,"Search (""Anterior Cruciate Ligament""[Mesh]) OR (((((((((((((Anterior cruciate ligament[Title/Abstract]) OR Anterior Cranial Cruciate Ligament[Title/Abstract]) OR Cranial Cruciate Ligament[Title/Abstract]) OR Cranial Cruciate Ligaments[Title/Abstract]) OR Cruciate Ligament, Cranial[Title/Abstract]) OR Cruciate Ligaments, Cranial[Title/Abstract]) OR Ligament, Cranial Cruciate[Title/Abstract]) OR Ligaments, Cranial Cruciate[Title/Abstract]) OR Cruciate Ligament, Anterior[Title/Abstract]) OR Anterior Cruciate Ligaments[Title/Abstract]) OR Cruciate Ligaments, Anterior[Title/Abstract]) OR Ligament, Anterior Cruciate[Title/Abstract]) OR Ligaments, Anterior Cruciate[Title/Abstract])",18212,09:50:32

#10,"Search ((((((((((((Anterior cruciate ligament[Title/Abstract]) OR Anterior Cranial Cruciate Ligament[Title/Abstract]) OR Cranial Cruciate Ligament[Title/Abstract]) OR Cranial Cruciate Ligaments[Title/Abstract]) OR Cruciate Ligament, Cranial[Title/Abstract]) OR Cruciate Ligaments, Cranial[Title/Abstract]) OR Ligament, Cranial Cruciate[Title/Abstract]) OR Ligaments, Cranial Cruciate[Title/Abstract]) OR Cruciate Ligament, Anterior[Title/Abstract]) OR Anterior Cruciate Ligaments[Title/Abstract]) OR Cruciate Ligaments, Anterior[Title/Abstract]) OR Ligament, Anterior Cruciate[Title/Abstract]) OR Ligaments, Anterior Cruciate[Title/Abstract]",16955,09:50:09

#9,"Search ""Anterior Cruciate Ligament""[Mesh]",9785,09:47:02
