# Supplementary material for: Remnant preservation technique versus standard technique for anterior cruciate ligament reconstruction: a meta-analysis of randomized controlled trials
Source: J Orthop Surg Res. 2018 Sep 12;13:231. doi: 10.1186/s13018-018-0937-4 (PMC6134761; doi:10.1186/s13018-018-0937-4)
Supplement: Supplementary file 2 — Embase. (DOCX 63 kb) [file 13018_2018_937_MOESM2_ESM.docx]

Embase

Session Results

.......................................................

No. Query Results Results Date

#17. #15 AND #16 203 24 Dec 2017

#16. 'remnant':ab,ti 22,157 24 Dec 2017

#15. #1 OR #2 OR #3 OR #4 OR #5 OR #6 OR #7 OR #8 OR 20,102 24 Dec 2017

#9 OR #10 OR #11 OR #12 OR #13 OR #14

#14. 'ligaments, anterior cruciate':ab,ti 2 24 Dec 2017

#13. 'ligament, anterior cruciate':ab,ti 15 24 Dec 2017

#12. 'cruciate ligaments, anterior':ab,ti 24 Dec 2017

#11. 'anterior cruciate ligaments':ab,ti 405 24 Dec 2017

#10. 'cruciate ligament, anterior':ab,ti 10 24 Dec 2017

#9. 'ligaments, cranial cruciate':ab,ti 1 24 Dec 2017

#8. 'ligament, cranial cruciate':ab,ti 24 Dec 2017

#7. 'cruciate ligaments, cranial':ab,ti 24 Dec 2017

#6. 'cruciate ligament, cranial':ab,ti 24 Dec 2017

#5. 'cranial cruciate ligaments':ab,ti 50 24 Dec 2017

#4. 'cranial cruciate ligament':ab,ti 704 24 Dec 2017

#3. 'anterior cranial cruciate ligament':ab,ti 6 24 Dec 2017

#2. 'anterior cruciate ligament':ab,ti 17,168 24 Dec 2017

#1. 'anterior cruciate ligament'/exp 10,331 24 Dec 2017

.......................................................
