# Supplementary material for: Remnant preservation technique versus standard technique for anterior cruciate ligament reconstruction: a meta-analysis of randomized controlled trials
Source: J Orthop Surg Res. 2018 Sep 12;13:231. doi: 10.1186/s13018-018-0937-4 (PMC6134761; doi:10.1186/s13018-018-0937-4)
Supplement: Supplementary file 3 — Cochrane. (DOCX 81 kb) [file 13018_2018_937_MOESM3_ESM.docx]

Search Name:

Date Run: 24/12/17 17:10:04.866

Description:

ID Search Hits

#1 MeSH descriptor: [Anterior Cruciate Ligament] explode all trees 755

#2 Anterior cruciate ligament:ti,ab,kw (Word variations have been searched) 1784

#3 Anterior Cranial Cruciate Ligament:ti,ab,kw (Word variations have been searched) 0

#4 Cranial Cruciate Ligament:ti,ab,kw (Word variations have been searched) 1

#5 Cranial Cruciate Ligaments:ti,ab,kw (Word variations have been searched) 1

#6 Cruciate Ligament, Cranial:ti,ab,kw (Word variations have been searched) 1

#7 Cruciate Ligaments, Cranial:ti,ab,kw (Word variations have been searched) 1

#8 Ligament, Cranial Cruciate:ti,ab,kw (Word variations have been searched) 1

#9 Ligaments, Cranial Cruciate:ti,ab,kw (Word variations have been searched) 1

#10 Cruciate Ligament, Anterior:ti,ab,kw (Word variations have been searched) 1784

#11 Anterior Cruciate Ligaments:ti,ab,kw (Word variations have been searched) 1784

#12 Cruciate Ligaments, Anterior:ti,ab,kw (Word variations have been searched) 1784

#13 Ligament, Anterior Cruciate:ti,ab,kw (Word variations have been searched) 1784

#14 Ligaments, Anterior Cruciate:ti,ab,kw (Word variations have been searched) 1784

#15 #1 or #2 or #3 or #4 or #5 or #7 or #8 or #9 or #10 or #11 or #12 or #12 or #14 1785

#16 remnant:ti,ab,kw (Word variations have been searched) 909

#17 #15 and #16 20
